# Trombose Ventricular Esquerda e Tromboembolismo Pulmonar em um Paciente de Covid-19 Assintomático

**DOI:** 10.36660/abc.20210590

**Published:** 2022-11-23

**Authors:** Natalia Lorenzo, Veronica Hernandez, Alvaro Montes, Fernando Rivero, Guillermo Reyes*, Rio Aguilar

**Affiliations:** 1 Hospital Universitario Infanta Cristina Cardiologia Parla Espanha Hospital Universitario Infanta Cristina - Cardiologia, Parla – Espanha; 2 Hospital Universitario de la Princesa Cardiologia e Cirurgia Cardíaca Madrid Espanha Hospital Universitario de la Princesa - Cardiologia e Cirurgia Cardíaca, Madrid – Espanha

**Keywords:** COVID-19/complicações, SARS-COV-2, Embolia Pulmonar, Disfunção do Ventrículo Esquerdo, Infarto do Miocárdio, Síndrome Respiratória Aguda Grave/complicações, Diagnóstico por Imagem/métodos

A COVID-19 (doença do coronavírus) é a síndrome associada à infecção síndrome respiratória aguda grave coronavírus 2 (SARS-CoV-2). Embora a insuficiência respiratória seja a característica mais aparente da doença, a trombose venosa e arterial são complicações bem reconhecidas. Sabe-se que pacientes de COVID-19 ativam várias respostas inflamatórias e coagulatórias sistêmicas que são vitais para a defesa do hospedeiro, mas podem levar a situações deletérias, principalmente para os pacientes internados em unidades de terapia intensiva. Este relato de caso apresenta múltiplos eventos embólicos com trombose do ventrículo esquerdo e coincidência de tromboembolismo pulmonar em um paciente com infecção por COVID-19 assintomática e sem doença cardiovascular preexistente.

## Carta Científica

Um homem de 48 anos chegou ao atendimento de emergência com dor abdominal e vômito com duração de 12 horas. O paciente teve um histórico de trombose venosa porto-esplênica dez anos antes da admissão, foi tratado por 6 meses com anticoagulação oral e considera-se que isso se devia a uma deficiência moderada de proteína C, que não foi confirmada em exames laboratoriais subsequentes. Os sinais vitais estavam normais; ele estava afebril e não houve achados significativos no exame físico, exceto por dor no lado esquerdo à apalpação. O teste de reação em cadeia da polimerase via transcriptase reversa (RT-PCR) para COVID-19 foi negativo. Os exames de sangue mostraram elevação significativa dos reagentes de fase aguda (proteína C reativa: 138,3 mg/L, fibrinogênio > 500 mg/dL e leucócitos 12,99 10^3/*μ*L). Os parâmetros de coagulação estavam dentro da faixa normal: o tempo de protrombina (TP) foi de 12,1 segundos, o tempo de tromboplastina parcial (PTT) foi de 36,3 segundos, a atividade de protrombina (AP) foi de 86%, a razão normalizada internacional (RNI) 1,08 e plaquetas 326.000/*μ*L.

**Figura 1:**
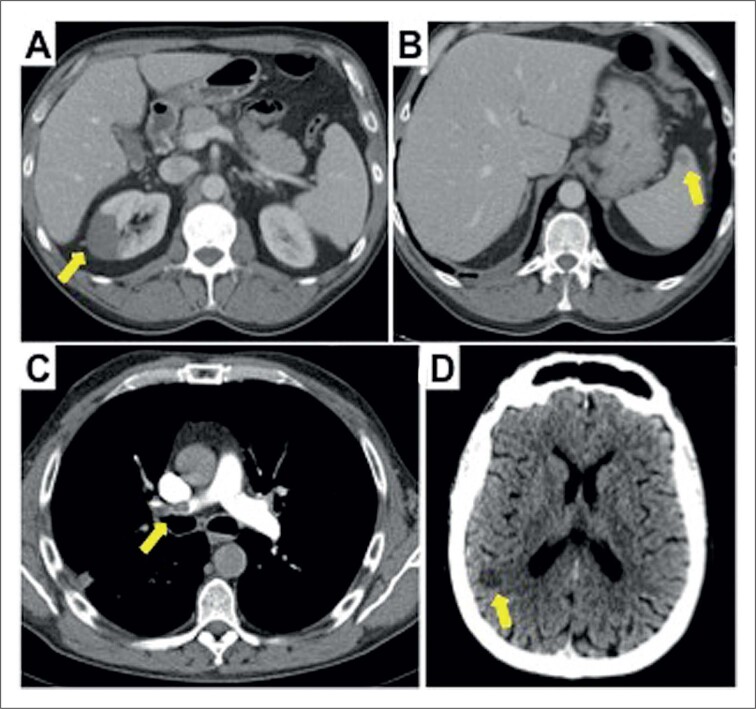
A) Tomografia computadorizada (TC) abdominal. Infarto do rim direito (seta). B) TC abdominal. Infarto no baço). C) TC cerebral. Lesão isquêmica na junção parieto-occipital direita (seta). D) TC do tórax. Defeito de enchimento originado na artéria pulmonar principal direita (seta).

**Figura 2:**
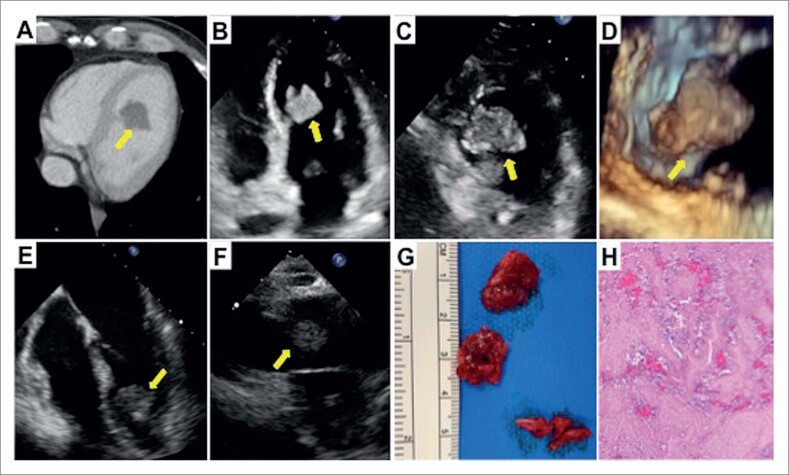
A) TC do tórax. Defeito de enchimento no ventrículo esquerdo (seta). B) Ecocardiograma transtorácico 2D. Vista de 4 eixos Massa hiperecogênica pedunculada (seta). C) Ecocardiograma transtorácico 2D. A massa (seta) em vista de eixo curto. D) Ecocardiograma transtorácico 3D. Massa ancorada no septo do ventrículo esquerdo (seta). E) Ecocardiograma transesofágico 2D. Vista de 4 câmaras. Massa ancorada no septo do ventrículo esquerdo (seta). F) Trombo no ramo pulmonar direito (seta). G) Peça cirúrgica retirada do ventrículo esquerdo. H) Anatomia patológica do trombo.

O ultrassom abdominal não revelou achados relevantes, e, portanto, foi realizada uma tomografia computadorizada (TC). A TC abdominal encontrou múltiplos infartos no rim direito (Figura 1A) e isolados no baço (Figura 1B). Além disso, observou-se um defeito de enchimento no ventrículo esquerdo (Figura 2A). O ecocardiograma transtorácico confirmou a presença de uma massa móvel hiperecogênica e homogênea (3,1 x 2 cm) com bordas regulares (Figuras 2A, 2B, 2C), ancorado ao terço intermediário do septo do ventrículo esquerdo (VE). A anatomia do VE estava normal, com dimensões normais e fração de ejeção normal sem anormalidades na movimentação da parede.

A TC do tórax também revelou tromboembolismo pulmonar, sem um efeito de enchimento originado na artéria pulmonar principal direito (Figura 1C), associado a infarto pulmonar no lóbulo superior direito; e a TC cerebral detectou uma lesão isquêmica subaguda na junção parieto-occipital (Figura 1D). Com o diagnóstico do tromboembolismo pulmonar e provavelmente de trombo no ventrículo esquerdo com múltiplas lesões embólicas, o paciente foi submetido a cirurgia cardíaca para retirar a massa. Embora o eletrocardiograma estivesse normal e as troponinas cardíacas estivessem dentro da faixa normal, foi realizada uma angiografia coronária invasiva no pré-operatório, que não revelou doença arterial coronariana aterosclerótico ou embolia coronária. O ecocardiograma transesofágico intraoperatório mostrou dilatação do tronco pulmonar, com a presença de uma imagem compatível com trombo no ramo pulmonar direito (Figura 2F). Além disso, a comunicação interatrial e o forame oval patente foram excluídos após uma ecografia com contraste com microbolhas. Foi realizado um novo teste RT-PCR para COVID-19 2 dias após a admissão hospitalar, com resultado positivo (variante Alfa [B.1.1.7], comumente chamada de variante britânica). Após alguns dias a presença da COVID-19 foi confirmada no IgG. O paciente não teve sintomas de infecção e nenhum momento. A maioria da massa pode ser retirada com cirurgia (Figura 2G), e a anatomia patológica confirmou que era um trombo (Figura 2H). A evolução subsequente foi favorável na anticoagulação com enoxaparina. Novos exames laboratoriais para avaliar a presença de uma coagulopatia mostraram deficiência leve de proteína C.

A presença de trombos cardíacos no ventrículo esquerdo é uma condição comum em pacientes com infarto do miocárdio (IM) (15-25%) e no surgimento de cardiomiopatia dilatada (até 36%) quando detectada com as modalidades de imagens ideais.^1,2^ Entretanto, há apenas relatos episódicos em VE com estrutura normal, mesmo na presença de uma trombofilia.^3–5^

A coagulopatia, na forma de trombose venosa e arterial, é uma das sequelas mais graves da infecção por SARS-CoV-2, e foi associada a resultados ruins. Relatórios de alta incidência de trombose, apesar do uso de anticoagulante em doses profiláticas e terapêuticas levantam perguntas sobre uma fisiopatologia exclusiva da COVID-19. As hipóteses propostas incluem uma resposta inflamatória gravemente aumentada que leva à trombo-inflamação, por mecanismos tais como as tempestades de citocina, ativação do complemento e endotelite.^6–9^ Também já se sugeriu que o próprio vírus tem a possibilidade de ativar a cascata de coagulação.^10^

Embora a trombose seja frequentemente vista no cenário de pacientes com COVID-19 que estão em um estágio crítico da doença, eventos tromboembólicos importantes são raros em pacientes assintomáticos e infecções leves.^11^ Entretanto, até onde sabemos, não há relatos anteriores de trombose em vários locais, incluindo trombos no VE, em pacientes completamente assintomáticos do ponto de vista da infecção sem doença cardiovascular preexistente. Acreditamos que, em nosso paciente, a presença de uma coagulopatia prévia teve um papel relevante nessa forma rara de apresentação em um paciente com COVID-19.
