# Supplementary material for: Trombose Ventricular Esquerda e Tromboembolismo Pulmonar em um Paciente de Covid-19 Assintomático
Source: Arq Bras Cardiol. 2022 Nov 23;119(6):996–8. [Article in Portuguese] doi: 10.36660/abc.20210590 (PMC9814811; doi:10.36660/abc.20210590)
Supplement: Translation

# Translation: Left Ventricular Thrombosis and Pulmonary Thromboembolism in an Asymptomatic Covid-19 Patient

**Authors:** Natalia Lorenzo, Veronica Hernandez, Alvaro Montes, Fernando Rivero, Guillermo Reyes*, Rio Aguilar

**Affiliations:** 1 Parla Spain Hospital Universitario Infanta Cristina - Cardiologia, Parla – Spain; 2 Madrid Spain Hospital Universitario de la Princesa - Cardiologia e Cirurgia Cardíaca, Madrid – Spain

**Keywords:** COVID-19/complications, SARS-COV-2, Pulmonary Embolism, Ventricular Dysfunction Left, Infarction, Myocardial, Severe Acute Respiratory Syndrome/complications, Diagnostic, Imaging/methods

COVID-19 (coronavirus disease) is the clinical syndrome associated with Severe Acute Respiratory Syndrome Coronavirus 2 (SARS-CoV-2) infection. Although respiratory failure is the most apparent feature of the disease, venous and arterial thrombosis are well-recognized complications. COVID-19 patients are known to activate multiple systemic coagulation and inflammatory responses that are vital for host defense but can lead to deleterious situations, mainly in those admitted to the intensive care units. We report here a case of multiple embolic events with left ventricular thrombosis and co-incidence of pulmonary thromboembolism in a patient with asymptomatic COVID-19 infection and no pre-existing cardiovascular disease.

## Research Letter

A 48-year-old-man presented to the emergency room with abdominal pain and vomiting of 12-hour duration. He had a history of porto-splenic venous thrombosis ten years before admission that was treated with 6 months of oral anticoagulation and felt to be due to moderate protein C deficiency, which was not confirmed in subsequent laboratory tests. Vital signs were normal, he was afebrile and no remarkable findings were found on physical examination except for pain on palpation in the left flank. The reverse transcriptase polymerase chain reaction (RT-PCR) test for COVID-19 was negative. Blood tests showed significant elevation of acute phase reactants (c-reactive protein: 138.3 mg/L, fibrinogen > 500mg/dL and leukocites 12.99 10^3/*μ*L). Coagulation parameters where in normal range: phrotrombin time (PT) 12.1 seconds, partial thromboplastin time (PTT) 36.3 seconds, prothrombin activity (PA) 86%, international normalized ratio (INR) 1.08 and platelets 326.000/*μ*L.

The abdominal ultrasound-scan did not show releant findings, so a computed tomography (CT) scan study was completed. CT abdominal scan found multiple infarcts in the right kidney (Figure 1A) and isolated in spleen (Figure 1B), furthermore, a filling defect in the left ventricle was observed (Figure 2A). Transthoracic echocardiogram confirmed the presence of a pedunculated hyperechogenic and homogeneous mobile mass (3.1x2cm) with regular borders (Figures 2A, 2B, 2C), anchored to the middle third of the septum of left ventricle (LV). Anatomy of LV was normal, with normal dimensions and normal ejection fraction without wall motion abnormalities.

**Figure 1:** A) Abdominal computed tomography (CT) scan. Infarct in the right kidney (arrow). B) Abdominal CT Scan. Infart in the spleen). C) Cerebral CT Scan. Ischemic lesion in the right parieto-occipital junction (arrow). D) Chest CT Scan. Filling defect originating from the right main pulmonary artery (arrow).

**Figure 2:** A) Chest computed tomography (CT) Scan. Filling defect in the left ventricle (arrow). B) 2D-Transthoracic echocardiogram. 4-axis-view. Pedunculated hyperechogenic mass (arrow). C) 2D-Transthoracic echocardiogram. The mass (arrow) in short-axis-view. D) 3D-Transthoracic echocardiogram. Mass anchored to the septum of left ventricle (arrow). E) 2D-Transesophageal echocardiogram. 4-chamber-view. Mass anchored to the septum of left ventricle (arrow). F) Thrombus in the right pulmonary branch (arrow). G) Surgical piece removed from the left ventricle. H) Pathological anatomy of the thrombus.

CT chest scan also revealed pulmonary thromboembolism, with a filling defect originating from the right main pulmonary artery (Figure 1C), associated with pulmonary infarction in the right upper lobe; and brain CT found a subacute ischemic lesion in the right parieto-occipital junction (Figure 1D). With the diagnosis of pulmonary thromboembolism and probably left ventricular thrombus with multiple embolic lesions, the patient underwent cardiac surgery to remove the mass. Although electrocardiogram was normal and cardiac troponins were in normal range, an invasive coronary angiogram was performed preoperatively, and revealed no atherosclerotic coronary artery disease or coronary embolism. Intraoperative transesophageal echocardiogram showed dilation of the pulmonary trunk, with the presence of an image compatible with thrombus in the right pulmonary branch (Figure 2F), in addition, interatrial communication and patent foramen ovale were ruled out after negative echo bubble study. 2 days after admission, a new RT-PCR test for COVID-19 was done, with a positive result (Alpha variant [B.1.1.7], commonly known as the British variant). A few days later the presence of COVID-19 IgG was confirmed. The patient had no infectious symptoms at any time. The majority of the mass could be removed with surgery (Figure 2G), and pathological anatomy confirmed that it was thrombus (Figure 2H). The subsequent evolution was favorable under anticoagulation with enoxaparin. New laboratory tests to assess the presence of a coagulopathy disorder showed mild protein C deficiency.

The presence of cardiac left ventricular thrombus is a relatively common condition in patients with myocardial infarction (MI) (15-25%) and in the setting of dilated cardiomyopathy (up to 36%) when detected with optimal imaging modalities.^1,2^ However, there are only anecdotal reports in structurally normal LV even in the presence of a thrombophilia.^3–5^

Coagulopathy, in the form of venous and arterial thrombosis, is one of the most severe sequelae of the SARS-CoV-2 infection, and has been associated with poor outcomes. Reports of a high incidence of thrombosis despite prophylactic and therapeutic dose anticoagulation raise question about a pathophysiology unique to COVID-19. Proposed hypotheses include a severely heightened inflammatory response that leads to thrombo-inflammation, through mechanisms such as cytokine storm, complement activation, and endothelitis.^6–9^ It has also been suggested that the virus itself can possibly activate the coagulation cascade.^10^

Though thrombosis is frequently seen in the setting of critically ill COVID 19 patients, major thromboembolic events are rare in patients with asymptomatic or mild infections.^11^ However, to the best of our knowledge, there are no previous reports of thrombosis in multiple locations, including LV thrombi, in a completely asymptomatic patient from the infectious point of view without pre-existing cardiovascular disease. We believe that in our patient, the presence of a previous coagulopathy, has played a relevant role in this rare form of presentation of a patient with COVID-19.
